# Morphology-based cytogenetic risk prediction in multiple myeloma from bone marrow smears

**DOI:** 10.3389/fonc.2026.1790130

**Published:** 2026-03-18

**Authors:** Minjie Gao, Xiao Yan, Lu Chen, Guifang Ouyang, Yangguang Liu, Shikun Chen

**Affiliations:** 1 Department of Hematology, The First Affiliated Hospital of Ningbo University, Ningbo, Zhejiang, China; 2 Department of Clinical Laboratory, Affiliated Jinhua Hospital, Zhejiang University School of Medicine, Jinhua, Zhejiang, China; 3 College of Finance and Information, Ningbo University of Finance & Economics, Ningbo, Zhejiang, China

**Keywords:** bone marrow smear, cytogenetics, multiple instance learning, multiple myeloma, plasma cell morphology, risk stratification

## Abstract

Cytogenetic abnormalities determine prognosis and treatment selection in multiple myeloma (MM). However, genetic testing via fluorescence *in situ* hybridization remains expensive and inaccessible in many clinical settings. Prior studies have established that specific chromosomal alterations correlate with distinct plasma cell morphological features. This suggests that visual analysis of bone marrow aspirate (BMA) smears could enable genetic risk prediction. We developed a computational framework that predicts high-risk cytogenetic status directly from digitized BMA smear images. Our approach employs DinoBloom, an established hematology-specific vision transformer pretrained on bone marrow and peripheral blood cell images, to extract morphological features from individual plasma cells. These embeddings are aggregated through attention-based multiple instance learning, which requires only patient-level labels rather than cell-by-cell annotations. The attention mechanism identifies which cells drive each prediction and links morphological phenotypes to genetic subtypes. Evaluated on a multi-center MM cohort with matched genetic results, the model achieved area under the curve values ranging from 0.76 to 0.85 across multiple cytogenetic markers. Attention maps highlighted plasmablastic features in high-risk predictions and mature plasma cell characteristics in standard-risk cases, consistent with established morphology-genetics correlations. This approach offers a rapid, cost-effective screening tool that could extend genetic risk stratification to resource-limited settings.

## 1 Introduction

Multiple myeloma (MM) is a clonal plasma cell malignancy characterized by the accumulation of abnormal plasma cells in the bone marrow (1). The disease exhibits marked genetic heterogeneity, and chromosomal abnormalities serve as the primary determinants of patient outcomes (2). The International Myeloma Working Group (IMWG) has established cytogenetic risk stratification as a cornerstone of treatment planning, with high-risk markers such as del(17p), t(4;14), and gain(1q) associated with inferior survival despite modern therapies (3). Conversely, the t(11;14) translocation identifies patients who may benefit from BCL2 inhibitors such as venetoclax (4). Accurate genetic characterization thus directly informs therapeutic decisions.

The current standard for cytogenetic assessment relies on fluorescence *in situ* hybridization (FISH). This technique requires specialized laboratory infrastructure, trained personnel, and turnaround times of three to seven days. The cost per test ranges from $500 to $2000 depending on the panel. These constraints limit access to genetic risk stratification in community oncology practices and resource-limited settings worldwide. A rapid, inexpensive screening method could extend precision medicine to underserved patient populations.

Plasma cell morphology offers an alternative window into the genetic landscape of MM. Garand et al. demonstrated that the t(11;14) translocation correlates with mature lymphoplasmacytoid morphology in over 90% of cases, whereas t(4;14) associates with immature cells that exhibit diffuse chromatin patterns (5). Plasmablastic morphology, characterized by large nuclei with prominent nucleoli and scant cytoplasm, constitutes an independent adverse prognostic factor (6). Quantitative morphological parameters including nuclear-to-cytoplasmic ratio, nuclear eccentricity, and chromatin texture also predict clinical outcomes (7). These observations suggest that visual features of plasma cells encode genetic information that trained observers—or computational algorithms—could potentially decode.

Recent advances in computational pathology have enabled automated analysis of bone marrow specimens. Matek et al. achieved human-level performance in blast cell recognition from acute myeloid leukemia (AML) smears (8), and subsequent work demonstrated accurate differentiation of over 20 bone marrow cell types (9). In parallel, weakly supervised methods such as multiple instance learning (MIL) have emerged as powerful tools for whole-slide image analysis. These approaches require only slide-level or patient-level labels rather than exhaustive cell-by-cell annotations (10, 11). Attention-based MIL architectures can identify which image regions drive classification decisions, thereby providing interpretable predictions (12). Foundation models pretrained on large hematological cell datasets now achieve state-of-the-art performance on downstream classification tasks (13). For AML, computational approaches have successfully predicted therapy-relevant genetic alterations directly from Pappenheim-stained bone marrow smears (14), including a recent study demonstrating AI-based prediction of RUNX1::RUNX1T1 abnormalities from bone marrow smear morphology (15). Xiao et al. developed a multi-scale AI platform that combines low-power and high-power lens fields for rapid batch screening of acute promyelocytic leukemia (16), illustrating the potential of automated morphological analysis at different magnification levels. In myelodysplastic syndromes (MDS), de Almeida et al. applied MIL with a multi-task objective to peripheral blood smears, predicting SF3B1 mutation status with an AUC of 0.90 and confirming disease-associated cytomorphologies through expert-assisted annotations (17). In MM, preliminary work has explored genetic prediction from bone marrow aspirate morphology (18) and from H&E-stained biopsy sections (19, 20), while Chen et al. demonstrated deep learning-based detection of circulating plasma cells in peripheral blood with high sensitivity, underscoring the growing role of AI in plasma cell identification across specimen types (21). However, a unified framework that leverages foundation models and attention-based MIL to predict multiple cytogenetic markers from MM bone marrow smears remains unexplored.

Here, we developed a computational pipeline that predicts high-risk cytogenetic status from digitized bone marrow aspirate smear images. Our approach first segments individual plasma cells from whole-slide images using established instance segmentation methods (22). We then extract morphological features via DinoBloom, an established hematology-specific vision transformer (13). These cell-level embeddings are aggregated through attention-based MIL, which identifies the specific cells that contribute to each genetic prediction. We evaluated the framework on a multi-center cohort of MM patients with matched FISH results. Our analysis reveals interpretable associations between morphological phenotypes and genetic subtypes that align with prior clinical observations.

## 2 Related work

### 2.1 Deep learning in hematological morphology

Computational methods for hematological diagnosis have evolved from handcrafted morphological features to deep learning approaches that achieve human-level performance. Early convolutional neural networks (CNNs) demonstrated accurate blast cell classification in AML (8), while subsequent architectures extended these capabilities to multi-class differentiation of over 20 bone marrow cell types (9). Acevedo et al. established baseline performance for peripheral blood cell recognition using CNNs trained on standardized image datasets (23). More recent work has shown that ensemble methods combining multiple deep learning models can further improve generalization across diverse hematological conditions (24). These advances have established digital pathology as a viable tool for routine hematological diagnostics.

### 2.2 Foundation models for pathology

The emergence of foundation models pretrained on large domain-specific datasets has addressed the limitations of ImageNet-based transfer learning in medical imaging. Chen et al. developed UNI, trained via self-supervised learning on over 100 million histopathology image tiles from more than 100,000 diagnostic slides spanning 20 tissue types (25). The model produces generalizable embeddings that achieve state-of-the-art performance across diverse classification and retrieval tasks. CONCH extends this paradigm by incorporating vision-language pretraining on 1.17 million histopathology image-caption pairs (26). Virchow, trained by Paige AI on 1.5 million whole-slide images (WSIs), demonstrated clinical-grade performance in pan-cancer detection with an area under the receiver operating characteristic curve (AUC) of 0.95 across 16 cancer types (27). Prov-GigaPath scaled to 1.1 billion parameters and achieved strong results across multiple pathology benchmarks (28). A recent benchmark of publicly available pathology foundation models found that CONCH and Virchow2 achieved the highest average AUCs across 31 evaluation tasks (29).

For hematological cell images specifically, Koch et al. developed DinoBloom, a vision transformer (ViT) trained via self-supervised learning on 380,000 bone marrow and peripheral blood cell images from 13 publicly available datasets (13). This model produces generalizable 768-dimensional embeddings that capture nuclear morphology, cytoplasmic features, and cell maturity indicators. When evaluated on downstream classification tasks, DinoBloom outperformed both ImageNet-pretrained models and general pathology foundation models, which demonstrates the value of hematology-specific pretraining.

### 2.3 Multiple instance learning for whole-slide analysis

Multiple instance learning provides a framework for weakly supervised analysis when only coarse-grained labels are available. In the context of whole-slide pathology, MIL formulates classification as a bag-level problem where each bag contains multiple instances (image patches or cells), but labels are assigned only to bags rather than individual instances (10). Ilse et al. introduced attention-based MIL (ABMIL), which learns to assign importance weights to each instance and aggregates them via weighted averaging (30). The attention weights provide interpretability by indicating which instances contribute most to the prediction. Lu et al. extended this approach with clustering-constrained attention multiple instance learning (CLAM), which incorporates instance-level clustering and contrastive learning to improve discrimination of heterogeneous tissue patterns (11). TransMIL further enhances MIL through transformer self-attention mechanisms that model long-range dependencies between instances and achieves AUCs of 0.93 on the Camelyon16 benchmark (12). Recent work has proposed attention entropy maximization (ACMIL) to prevent attention collapse and improve generalization (31), while MamMIL leverages state space models to reduce memory requirements by 65–70% compared to transformer-based approaches (32).

### 2.4 Genetic prediction from morphology

The hypothesis that genetic alterations manifest in observable cellular morphology has driven research on mutation prediction from pathology images. Coudray et al. demonstrated that CNNs trained on lung adenocarcinoma histopathology images could predict six commonly mutated genes with AUCs ranging from 0.73 to 0.86 (33). Kather et al. subsequently showed that this approach generalizes across multiple cancer types for microsatellite instability detection and other biomarkers (34).

In hematological malignancies, Eckardt et al. trained a CNN to detect AML and predict NPM1 mutation status directly from bone marrow smears, achieving an AUC of 0.92 for mutation prediction (35). For MM specifically, Mason et al. employed artificial intelligence to analyze 2-D and 3-D bone marrow microenvironment features from H&E-stained tissue biopsies to identify cytogenetic subtypes (20). However, this work focused on tissue biopsies rather than cytological smears, which offer superior single-cell resolution and preserve finer morphological detail. Preliminary work by Bermejo-Peláez et al. explored genetic prediction from bone marrow aspirate smears but was limited to a single institution and did not leverage foundation models (18). Terebelo et al. applied deep learning to predict t(11;14) from hematoxylin and eosin (H&E) samples (19). No prior study has combined hematology-specific foundation models, attention-based MIL, and multi-task learning to predict multiple cytogenetic markers from MM bone marrow smears.

### 2.5 Cell segmentation and explainability

Accurate extraction of individual cells from bone marrow smears requires robust instance segmentation. StarDist employs star-convex polygon representations to detect and segment cells with approximately convex shapes (36). Cellpose uses a gradient flow-based approach that can handle more varied cell morphologies without requiring shape priors (37). The SegPC-2021 challenge specifically addressed plasma cell segmentation in MM bone marrow images, with winning solutions achieving Dice coefficients above 0.90 (22).

Clinical adoption of computational pathology tools depends on model interpretability. Attention mechanisms in MIL architectures provide inherent interpretability by indicating which cells or regions drive each prediction. Complementary explainability methods include Gradient-weighted Class Activation Mapping (Grad-CAM), which highlights image regions contributing to CNN predictions (38), and SHAP, which quantifies feature importance based on game-theoretic principles (39). In the context of genetic prediction from morphology, these techniques can reveal whether models focus on biologically meaningful features rather than artifacts or batch effects.

## 3 Materials and methods

**Figure 1:**
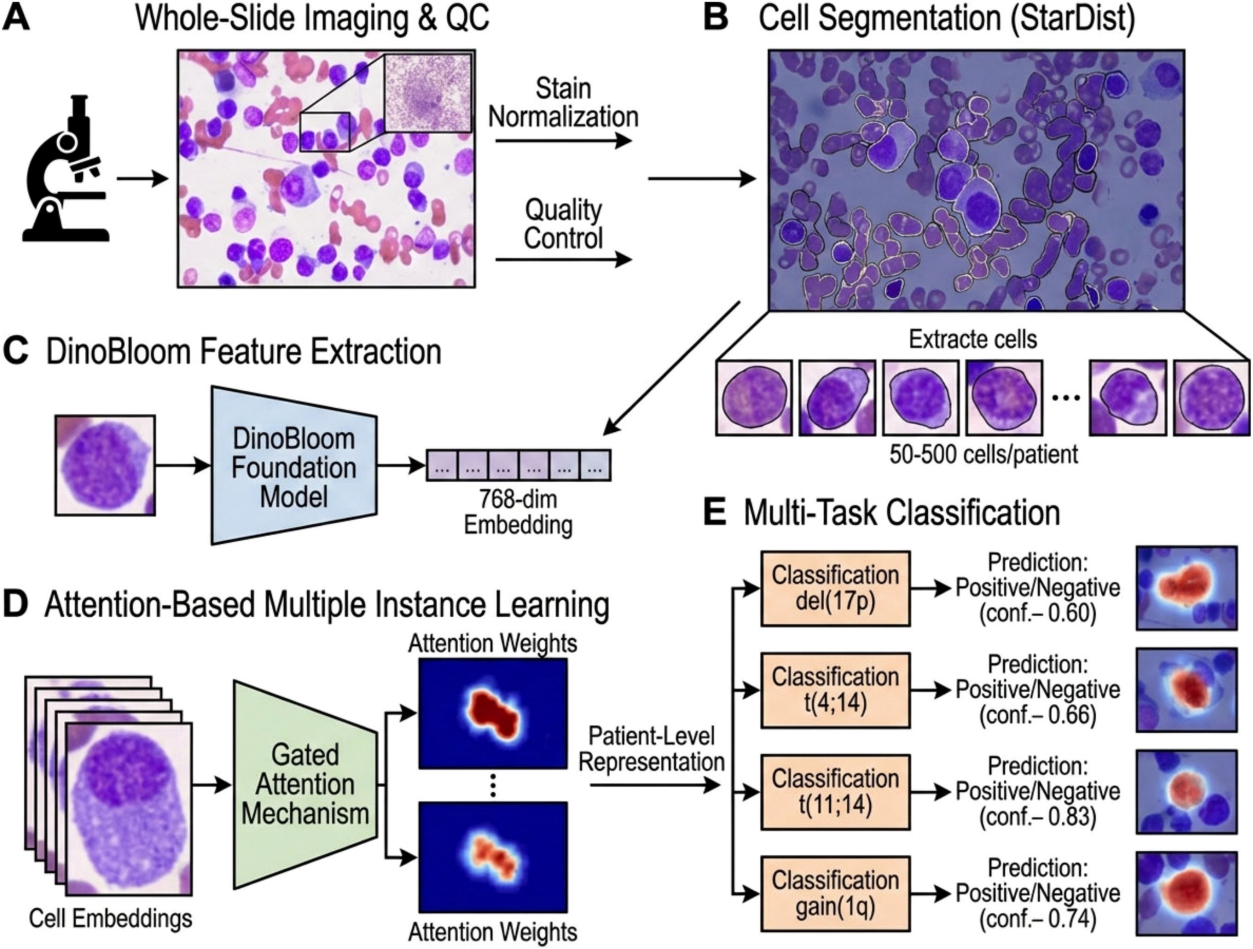
Computational pipeline for cytogenetic risk prediction from bone marrow aspirate smears. The framework comprises five stages: **(A)** Whole-slide imaging of Wright-Giemsa stained BMA smears followed by stain normalization and quality control. **(B)** Instance segmentation using StarDist (raw detections shown; non-plasma cells such as red blood cells are removed by subsequent size, nuclear-to-total-area ratio, and confidence score filtering; see Section 3.2). **(C)** Feature extraction via DinoBloom foundation model, producing 768-dimensional embeddings for each cell. **(D)** Attention-based multiple instance learning with gated attention mechanism that weights cell contributions and aggregates embeddings to patient-level representation. **(E)** Multi-task classification heads predicting del(17p), t(4;14), t(11;14), and gain(1q) simultaneously from shared representation. Attention weights enable identification of morphologically diagnostic cells (highlighted in red) that drive predictions for specific genetic subtypes. This end-to-end framework operates on patient-level FISH labels without cell-by-cell annotations, which addresses the impracticality of manual labeling at scale.

We developed a computational framework that integrates hematology-specific foundation models with attention-based multiple instance learning to predict cytogenetic risk directly from bone marrow aspirate smears. This approach represents the first application of DinoBloom embeddings to MM cytology, enables simultaneous multi-task prediction of multiple high-risk markers, and provides interpretable attention maps that link plasma cell morphology to genetic subtypes. The complete pipeline (**Figure 1**) proceeds from whole-slide image preprocessing through cell segmentation, foundation model feature extraction, attention-based aggregation, and multi-task classification with explainability analysis.

### 3.1 Study cohort and data acquisition

This retrospective study included patients diagnosed with MM according to International Myeloma Working Group criteria (40) at The First Affiliated Hospital of Ningbo University (Ningbo 315010, Zhejiang Province, China) in 2025. Eligible patients had bone marrow aspirate smears and corresponding FISH results available for analysis. The study protocol was approved by the institutional review board, and patient consent was waived for this retrospective analysis of clinically acquired data. Clinical variables including age, sex, International Staging System (ISS) stage (2), and treatment history were extracted from electronic medical records. All digitized whole-slide images and associated metadata were organized in the MM/directory structure.

Cytogenetic analysis was performed via FISH on CD138-enriched plasma cells from bone marrow aspirates. The standard panel included probes for del(17p), t(4;14), t(11;14), gain(1q), and t(14;16). A marker was considered positive when the percentage of abnormal cells exceeded laboratory-specific cutoff thresholds (typically 10–20% for translocations and 20–30% for deletions and gains). All FISH testing was performed in Clinical Laboratory Improvement Amendments (CLIA)-certified laboratories according to standard protocols.

Bone marrow aspirate smears were prepared immediately following sample collection and stained with Wright-Giemsa or May-Grunwald-Giemsa¨ according to institutional protocols. Slides were digitized using whole-slide scanners (Aperio AT2 or Hamamatsu NanoZoomer) at 40× magnification, yielding images with resolution of approximately 0.25 *µ*m per pixel. Digitized images were stored in standard whole-slide imaging formats (SVS, NDPI) and converted to pyramidal TIFF for processing. Slide quality was assessed by two experienced laboratory technicians who independently reviewed each digitized image. Slides were excluded if plasma cell content was below 10%—the minimum threshold for MM diagnosis per IMWG criteria (40) and for yielding sufficient plasma cells for MIL analysis. Slides were also excluded if artifacts (crush artifacts, air-drying artifacts, staining precipitate, or ink marks) affected more than 20% of the cellular zone, or if staining was too faint or too dark to resolve nuclear chromatin detail. Discrepancies between reviewers were resolved by consensus. Of 3,240 digitized images, 263 (8.1%) were excluded, yielding 2,977 slides for analysis. For each slide, analysis was restricted to the optimal cellular zone (monolayer region between the thick body and feathered edge) where cells are well-spread and non-overlapping, following standard practice for BMA morphological assessment.

Patients were randomly assigned to training (60%), validation (20%), and test (20%) sets using stratified splitting to maintain similar prevalence of each cytogenetic marker across subsets. Patient-level splitting ensured that all images from a given patient appeared in only one subset, preventing data leakage. The final cohort comprised 720 patients with 3,240 digitized whole-slide images (3–6 smears per patient, as multiple slides are routinely prepared from a single bone marrow aspiration to ensure adequate material; 2,977 after quality control) and a total of 182,943 individual plasma cells extracted for analysis.

### 3.2 Image preprocessing and cell segmentation

Wright-Giemsa staining exhibits variability across institutions, staining batches, and scanner models. To mitigate these effects, we applied stain normalization using the Macenko method (41), which identifies two principal stain vectors in optical density space via singular value decomposition. Although originally described for H&E stains, the Macenko method is stain-agnostic: it decomposes any bi-chromatic stain into its two dominant color components. For Wright-Giemsa and May-Grünwald-Giemsa (Romanowsky-type stains), these correspond to the basophilic component (azure B/methylene blue) and the eosinophilic component (eosin Y). A reference image with representative staining characteristics was selected from the training set based on median color statistics. Each whole-slide image was normalized to match this reference distribution, and morphological detail was preserved. Normalized images underwent a two-stage quality control (QC) process. First, automated filters excluded blurred regions (Laplacian variance *<* 100), intensity outliers (mean pixel intensity outside the 5th–95th percentile range of the reference image), and regions with low cell density (*<*5 nucleated cells per 200×200 *µ*m tile, estimated via Otsu thresholding on the basophilic channel). Second, a manual review by the same two laboratory technicians (see above) identified remaining artifacts not captured by automated filters, including ink marks and staining precipitate. Regions failing either stage were excluded from downstream analysis.

Individual plasma cells were segmented using StarDist (36), a deep learning-based instance segmentation method optimized for star-convex objects such as nucleated cells. The StarDist model was pretrained on the SegPC-2021 dataset (22), which comprises over 20,000 annotated plasma cells from MM bone marrow smears, and fine-tuned on a subset of 1,200 manually annotated cells from 30 patients drawn exclusively from the training split. Fine-tuning used the Adam optimizer with learning rate 3×10^−4^, batch size 8, and 50 epochs with early stopping (patience 10 epochs) based on validation Dice score on a held-out 20% of the annotated cells. Critically, the fine-tuning annotations were used only for segmentation model training and were not included as labeled instances in the downstream MIL pipeline; the MIL model relied solely on patient-level FISH labels. The model outputs instance masks that identify individual cell boundaries along with confidence scores. Since StarDist inherently provides instance-level segmentation via star-convex polygon fitting and non-maximum suppression, no additional instance separation was required. Post-processing was limited to removal of small spurious detections (*<*200 pixels) and filtering based on circularity (*>*0.6) and eccentricity (*<*0.85) to exclude artifacts and cell fragments. These thresholds were determined empirically by examining the shape distributions of 500 manually verified plasma cells from the training set, including atypical morphologies (plasmablastic, bi-nucleated). Manual review of a random sample of 200 excluded objects confirmed that 94% were artifacts (cell debris, overlapping clusters, staining precipitate) rather than intact plasma cells. The thresholds are sufficiently permissive to retain diagnostically relevant atypical cells: plasmablastic cells, despite their large size, maintain circularity values typically above 0.7, and bi-nucleated plasma cells yielded eccentricity values below 0.80 in our annotated set.

Within each cell instance mask, the nucleus was segmented from the cytoplasm using Otsu thresholding applied to the basophilic channel obtained from Macenko stain deconvolution. The nucleus corresponds to the deeply basophilic region (dark blue/purple under Wright-Giemsa staining), which is well separated from the lighter cytoplasm in optical density space. The nucleus centroid was computed as the intensity-weighted center of mass of the nuclear mask, and the nuclear boundary was refined by morphological closing (3×3 kernel) to fill small gaps.

For each segmented cell, we extracted a square bounding box centered on the nucleus centroid and expanded to 128×128 pixels to include surrounding cytoplasm. Centering on the nucleus rather than the cell centroid ensures that nuclear features—chromatin texture, nucleolar prominence, and nuclear shape—are fully captured within the patch, which is important because plasma cells characteristically exhibit eccentric nuclei and centering on the cell centroid risks truncating nuclear detail. Patches were padded with background color if the bounding box extended beyond the image boundary. Only cells with confidence scores exceeding 0.7 and meeting morphological criteria (area 500–5000 pixels, nuclear-to-total-area ratio 0.2–0.8) were retained. These filters effectively exclude non-plasma cells: red blood cells (diameter ∼7 *µ*m, ∼50–80 pixels at 40×) fall below the 500-pixel area threshold, and their lack of a nucleus yields nuclear-to-total-area ratios near zero, well outside the 0.2–0.8 range. Other nucleated cells such as granulocytes and lymphocytes are largely excluded by the combination of the plasma cell-specific StarDist model (trained on SegPC-2021 annotations) and the morphological size and shape filters. This resulted in a median of 243 cells per patient (interquartile range [IQR]: 186–314; range: 68–823), with the distribution of bag sizes varying based on aspirate cellularity and plasma cell burden.

### 3.3 Foundation model feature extraction and multi-task attention framework

Our framework leverages DinoBloom (13), an established hematology- and leukocyte-specific vision transformer pretrained via self-supervised learning on bone marrow and peripheral blood cell images (see Section 2.2 for details). Unlike prior work employing ImageNet-pretrained models or H&E biopsy images, DinoBloom’s domain-specific pretraining on cytological specimens produces 768-dimensional embeddings that capture morphological features directly relevant to plasma cell differentiation states and genetic subtypes. The model employs a DINO (self-distillation with no labels) training objective optimized for single-cell hematology images. We used the publicly available ViT-B/16 checkpoint from HuggingFace (1aurent/dinobloom-vit-base), which accepts 224×224 pixel RGB inputs. Each 128×128 cell patch was resized to 224×224 via bilinear interpolation and normalized to [0,1] intensity range. Critically, the DinoBloom encoder was frozen during training to preserve pretrained representations. This reduced trainable parameters and prevented overfitting on the relatively small labeled cohort.

We formulated the prediction task as multiple instance learning, where each patient corresponds to a bag *B_i_*= {**x**_1_,**x**_2_*,…*,**x***_N_*} containing *N* cell instances. The bag label *y_i_*∈ {0,1} indicates presence or absence of a cytogenetic marker based on FISH results. Each instance is represented by its DinoBloom embedding **h***_j_*∈ R^768^. The MIL framework predicts the bag label without instance-level annotations, which addresses the impracticality of manually labeling thousands of cells per patient. Each cytogenetic marker was treated as an independent binary classification task. Patients with co-occurring abnormalities (n = 125, 17.4% of the cohort) were labeled as positive for all relevant markers simultaneously; for example, a patient harboring both del(17p) and t(11;14) contributed as a positive example to both classifiers. Patients negative for all four target markers (n = 270, 37.5%) served as negative examples for every task. This formulation allows the model to output simultaneous predictions for multiple markers in a single forward pass, reflecting the clinical reality that co-occurring abnormalities are common in MM.

We employed attention-based aggregation to weight the contribution of each cell to the patient-level prediction. Following the ABMIL architecture (30), attention scores were computed via a gated attention mechanism (Equation 1):

(1) $$a_{j}=\frac{\exp\;(w^{T}(\tanh\;(Vh_{j}^{T})⊙\sigma (Uh_{j}^{T})))}{\sum_{k=1}^{N}\exp\;(w^{T}(\tanh\;(Vh_{k}^{T})⊙\sigma (Uh_{k}^{T})))}$$

where $V,U\in {\mathbb{R}}^{256\times 768}$ are learnable projection matrices, $w\in {\mathbb{R}}^{256}$ is a learnable weight vector, tanh is the hyperbolic tangent activation, σ is the sigmoid activation, and ⊙ denotes element-wise multiplication. The gating mechanism allows the model to learn which features are relevant for attention computation. The bag-level representation is obtained via weighted aggregation (Equation 2):

(2) $$z_{i}=\sum_{j=1}^{N}a_{j}h_{j}$$

The aggregated representation $z_{i}\in {\mathbb{R}}^{768}$ serves as input to task-specific classification heads for each cytogenetic marker. Our framework employs multi-task learning that simultaneously predicts del(17p), t(4;14), t(11;14), and gain(1q) from a single shared attention module, which enables the model to learn common morphological patterns across related genetic alterations while maintaining marker-specific discrimination. This contrasts with prior approaches that trained separate models for each marker and missed opportunities for transfer learning across correlated cytogenetic features. Each marker is predicted via a dedicated linear classifier (Equation 3):

(3) $${\hat{y}}_{i,t}=\sigma (W_{t}z_{i}+b_{t})$$

where $W_{t}\in {\mathbb{R}}^{768}$ and $b_{t}\in \mathbb{R}$ are learnable parameters for marker t, and σ is the sigmoid activation. The multi-task loss aggregates binary cross-entropy across all markers (Equation 4):

(4) $$L=\frac{1}{\left|T\right|}\sum_{t\in T}-[y_{i,t}\text{log }({\hat{y}}_{i,t})+(1-y_{i,t})\text{log }(1-{\hat{y}}_{i,t})]$$

where T denotes the set of cytogenetic markers and the loss is computed only for markers with available FISH results (missing labels were excluded from the summation). Uniform weighting ( $\lambda_{t}=1$) was applied across tasks, as preliminary experiments with prevalence-based weighting did not improve performance. This multi-task architecture enables efficient simultaneous risk assessment at diagnosis and outputs predicted probabilities for all four markers from a single forward pass.

### 3.4 Model training and evaluation

The model was implemented in PyTorch 2.0 and trained on NVIDIA RTX 4090 GPU with 48 GB memory. Training employed the AdamW optimizer (42) with learning rate 2×10^−4^, weight decay 1×10^−4^, and batch size 16 bags (patients). The learning rate was reduced by a factor of 0.5 when validation loss plateaued for more than 5 epochs. Gradient clipping with maximum norm 1.0 prevented instability during training. Models were trained for a maximum of 100 epochs with early stopping based on validation AUC (patience 15 epochs). The best model checkpoint was selected based on average AUC across all markers on the validation set.

Data augmentation was applied at the cell level during training. Each cell patch underwent random horizontal and vertical flipping (probability 0.5), random rotation (uniform ±180°), and color jitter (brightness ±0.1, contrast ±0.1, saturation ±0.1). A total of 87 patients (12.1%) yielded more than 500 cells after filtering. For these patients, we randomly sampled 500 cells per epoch to reduce memory consumption and training time; this introduced additional stochastic regularization. At inference, all detected cells were used without subsampling. Dropout (rate 0.2) was applied after the attention module and within classification heads.

Class imbalance was addressed through two complementary strategies. First, we applied oversampling during batch construction: patients positive for rare markers [del(17p), t(4;14)] were sampled with higher probability so that each training batch contained approximately balanced positive and negative examples per marker. Second, we used focal loss (43) with focusing parameter *γ* = 2 and class weights proportional to the inverse square root of marker prevalence. This formulation down-weights easy-to-classify examples and focuses learning on hard cases, particularly important for rare markers such as del(17p) (prevalence ∼10%).

Model performance was evaluated on the held-out test set using area under the receiver operating characteristic curve (AUC) as the primary metric. For each marker, we computed sensitivity, specificity, precision, recall, F1-score, and balanced accuracy at the threshold that maximized the Youden index (sensitivity + specificity - 1) on the validation set; this threshold was then applied to the test set for all reported metrics. Confidence intervals (95%) for AUC were estimated via bootstrapping with 1000 iterations. Statistical significance of performance differences between models was assessed using the DeLong test for paired AUC comparisons.

We compared the proposed approach (DinoBloom embeddings with attention-based MIL) against several baselines: (1) mean pooling of DinoBloom embeddings followed by logistic regression, (2) max pooling of DinoBloom embeddings, (3) attention-based MIL with ImageNet-pretrained ResNet50 features, (4) attention-based MIL with UNI embeddings (25), a general histopathology foundation model pretrained on over 100 million pathology image tiles, (5) attention-based MIL with CONCH embeddings (26), a vision-language pathology foundation model pretrained on 1.17 million histopathology image-caption pairs, and (6) logistic regression (LR) on handcrafted morphological features extracted using CellProfiler. Ablation studies evaluated the contribution of individual components including attention mechanism, multi-task learning, and stain normalization.

Subgroup analyses assessed performance stratification by institution, scanner model, patient age (*<*65 vs ≥65 years, the standard transplant-eligibility threshold in MM clinical practice), disease stage (ISS I/II vs III), and bag size tertiles. These analyses evaluated model robustness and identified potential sources of performance heterogeneity. Stratified analyses used the same evaluation metrics as the primary analysis, with statistical testing (Mann-Whitney U test) to detect significant differences between subgroups.

### 3.5 Explainability and interpretability analysis

**Figure 2:**
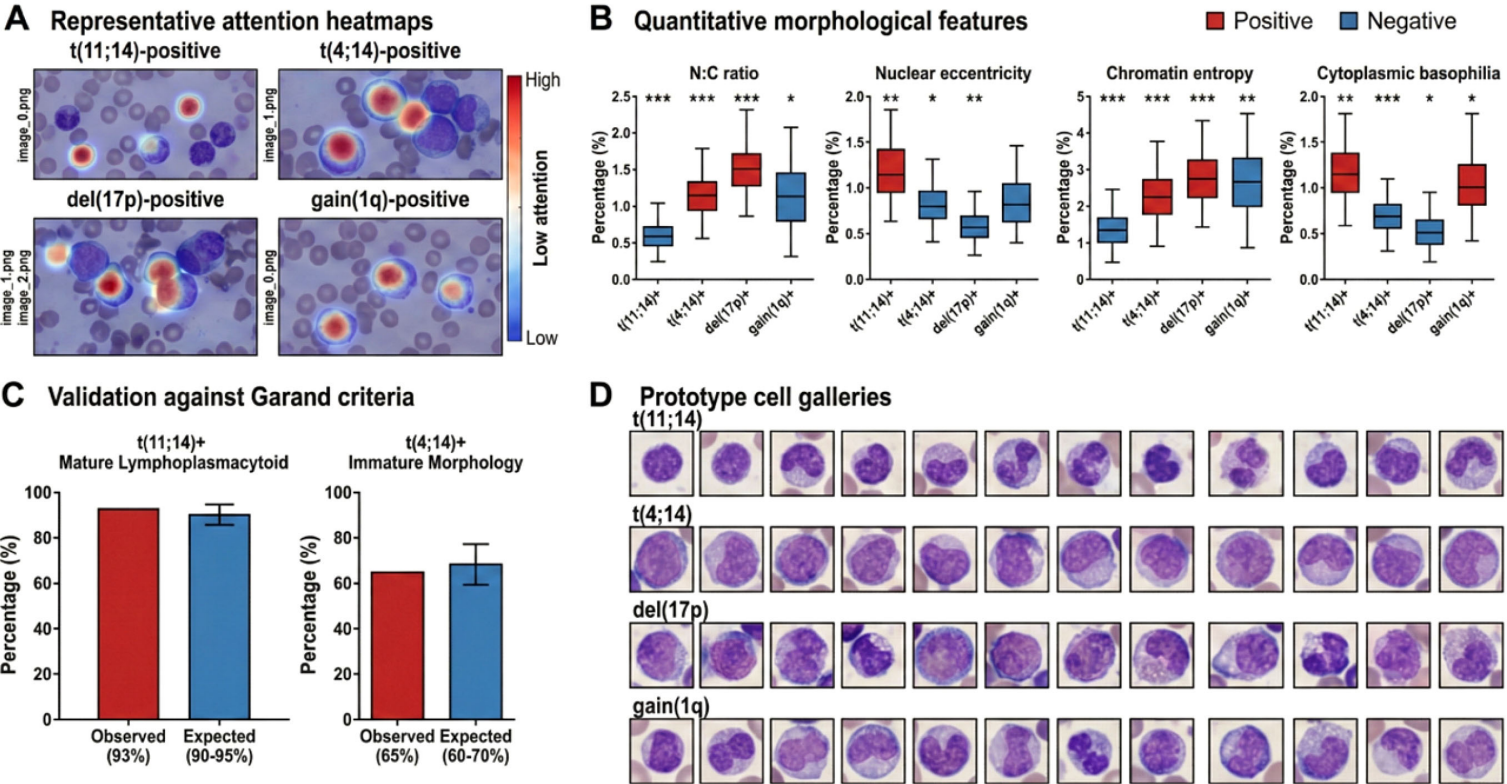
Attention-based identification of morphologically diagnostic cells and validation against known morphology-genetics correlations. **(A)** Representative attention heatmaps for four genetic subtypes overlaid on bone marrow aspirate smears. Although the model processes individual cell patches independently, attention weights were mapped back to each cell’s spatial coordinates on the original smear for visualization. High-attention cells (red) drive predictions, while low-attention cells (blue) contribute minimally. t(11;14)-positive cases show attention focused on mature lymphoplasmacytoid cells with abundant cytoplasm, whereas t(4;14)-positive cases highlight immature cells with high nuclear-tocytoplasmic ratios. **(B)** Quantitative morphological features extracted from high-attention cells (top 20% by attention score) compared between positive and negative cases for each marker. Box plots show N:C ratio, nuclear eccentricity, chromatin entropy, and cytoplasmic basophilia. Statistical significance indicated by *(p *<* 0.05), **(p *<* 0.01), ***(p *<* 0.001, Welch’s t-test with Bonferroni correction). **(C)** Validation against Garand criteria: percentage of high-attention cells in t(11;14)-positive cases exhibiting mature lymphoplasmacytoid morphology (93% observed vs 90–95% expected from literature), and percentage in t(4;14)-positive cases showing immature morphology (65% observed vs 60–70% expected). **(D)** Prototype cell galleries displaying the 20 cells with highest attention scores from representative positive cases for each marker, demonstrating learned morphological signatures: t(11;14) selects small mature cells, t(4;14) selects plasmablasts, del(17p) associates with high-risk plasmablastic features, and gain(1q) shows intermediate morphology. This figure establishes that attention mechanisms capture clinically validated morphology-genetics relationships, providing biological interpretability and supporting clinical trust in model predictions.

Our attention-based framework provides interpretability through automatic identification of plasma cells that contribute to each genetic prediction, which enables validation against established morphology-genetics correlations and facilitates discovery of previously uncharacterized associations. For each prediction, we extracted the attention weights {*a*_1_*,a*_2_*,…,a_N_*} assigned to individual cells. Cells in the top 20% of attention scores were designated as informative instances driving the prediction. These high-attention cells were visualized by overlaying attention heatmaps on the original whole-slide images, with color intensity proportional to attention weight (blue: low, red: high). Spatial clustering of high-attention cells was assessed using density-based spatial clustering (DBSCAN) to identify whether diagnostically relevant cells were localized to specific regions. **Figure 2** illustrates how attention patterns differ across genetic subtypes and correlate with known morphological features.

To link attention patterns with morphological features, we extracted quantitative characteristics from high-attention cells using automated image analysis. Nuclear features included area, perimeter, eccentricity (ratio of major to minor axis), and circularity (4*π*× area/perimeter^2^). The nuclear-to-cytoplasmic (N:C) ratio was computed as the ratio of nuclear area to total cell area. Chromatin texture was quantified via gray-level co-occurrence matrix (GLCM) features including entropy, homogeneity, contrast, and correlation, computed on the basophilic channel obtained from Macenko stain deconvolution to isolate chromatin specific signal. Cytoplasmic basophilia was measured as the mean optical density in the basophilic channel within cytoplasmic regions. These features were extracted using OpenCV and scikit-image libraries. We compared morphological features between cells from patients with versus without each cytogenetic marker, focusing on high-attention cells to identify features associated with specific genetic subtypes. Statistical comparisons employed Welch’s t-test for two-group comparisons (normal distributions) or Mann-Whitney U test (non-normal distributions), with Bonferroni correction for multiple testing. Effect sizes were quantified using Cohen’s d. We validated our findings against established morphology-genetics correlations from Garand et al. (5), specifically examining whether t(11;14)-positive cases exhibited mature lymphoplasmacytoid features (small size, abundant cytoplasm, clock-face chromatin) and whether t(4;14)-positive cases showed immature morphology (high N:C ratio, diffuse chromatin, prominent nucleoli). This validation step confirms that the model learns biologically meaningful associations rather than technical artifacts or batch effects.

For interpretability assessment, we generated prototype cell galleries by retrieving the 20 cells with highest attention scores from positive and negative cases for each marker. These galleries provided visual summaries of morphological patterns learned by the model. To validate clinical relevance, two board-certified hematopathologists independently reviewed attention heatmaps for 50 randomly selected cases (balanced across markers and predictions). Each pathologist rated the biological plausibility of highlighted cells on a 5-point Likert scale (1: not plausible, 5: highly plausible) and provided qualitative feedback on agreement between AI-identified cells and diagnostically relevant features. As a control, pathologists also rated galleries of 20 randomly sampled cells from the same patients, presented in randomized order and blinded to the selection method (attention-based vs. random). This controlled design enables direct comparison of plausibility ratings between attention-selected and randomly selected cell galleries. Inter-rater reliability was quantified using Cohen’s kappa, and agreement between AI and pathologist annotations was assessed via percentage overlap of high-attention versus manually annotated cells.

## 4 Results

### 4.1 Cohort characteristics and data overview

The final cohort comprised 720 patients diagnosed with MM at The First Affiliated Hospital of Ningbo University between 2020 and 2025. Patient demographics and clinical characteristics are summarized in **Table 1**. The median age at diagnosis was 64 years (range: 40–85 years), with a slight male predominance (52.6%, 379/720). Disease staging according to the International Staging System showed 181 patients (25.1%) with ISS stage I, 245 patients (34.0%) with ISS stage II, and 294 patients (40.8%) with ISS stage III, reflecting a cohort enriched for advanced disease. All patients underwent cytogenetic evaluation via FISH on CD138-enriched plasma cells, with complete results available for del(17p), t(4;14), t(11;14), and gain(1q).

**Table 1:** Cohort characteristics (N = 720).

Characteristic	Value
Demographics	
Age, years	64 (58–71); range 40–85
Male/Female	379 (52.6%)/341 (47.4%)
ISS Stage	
I/II/III	181 (25.1%)/245 (34.0%)/294 (40.8%)
Cytogenetics (FISH)	
del(17p)/t(4;14)	76 (10.6%)/96 (13.3%)
t(11;14)/gain(1q)	133 (18.5%)/288 (40.0%)
Any abnormality	450 (62.5%)
High-risk^†^	160 (22.2%)
Imaging Data	
WSI images (after QC)	3,240 (2,977; 91.9%)
Total plasma cells	182,943
Cells/patient	243 (186–314); range 68–823
Data Split	
Train/Val/Test	432 (60%)/144 (20%)/144 (20%)

^†^del(17p) and/or t(4;14).

Values: median (IQR) or n (%).

**Figure 3:**
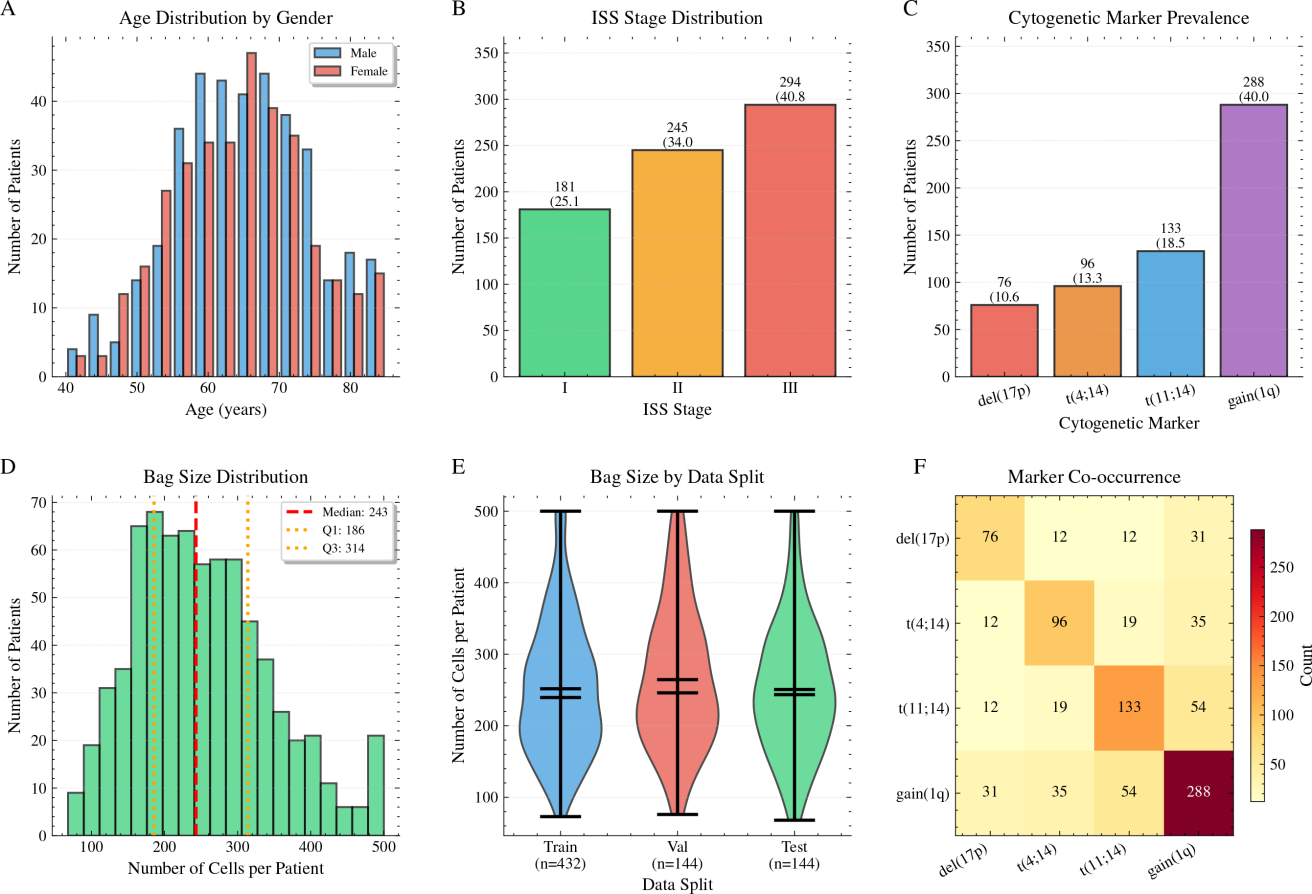
Cohort characteristics and data overview (N = 720). **(A)** Cytogenetic marker prevalence showing gain(1q) as most common (40.0%), followed by t(11;14) (18.5%), t(4;14) (13.3%), and del(17p) (10.6%). **(B)** Bag size distribution (cells per patient) with median 243 cells (IQR: 186–314), which indicates sufficient instances for robust MIL training. **(C)** Bag size comparison across training, validation, and test splits demonstrating comparable distributions, which confirms successful stratification. **(D)** Age distribution stratified by gender, with similar medians for male and female patients. **(E)** ISS stage distribution showing enrichment for advanced disease: stage III (40.8%), stage II (34.0%), and stage I (25.1%). **(F)** Summary statistics table presenting demographics, cytogenetic markers, imaging data, and cell counts.

Cytogenetic abnormalities were detected in 450 of 720 patients (62.5%), with multiple concurrent alterations present in 125 patients (17.4%). The prevalence of individual markers (**Figure 3C**) reflected expected distributions from the literature: gain(1q) was the most common abnormality, detected in 288 patients (40.0%), followed by t(11;14) in 133 patients (18.5%), t(4;14) in 96 patients (13.3%), and del(17p) in 76 patients (10.6%). Marker co-occurrence patterns (**Figure 3F**) revealed that gain(1q) frequently co-occurred with other abnormalities, consistent with its role as a secondary event in MM pathogenesis. High-risk cytogenetics, defined as presence of del(17p), t(4;14), or both, were identified in 160 patients (22.2%). The observed prevalence rates align closely with published estimates (3), which confirms that our cohort represents a typical MM population suitable for developing generalizable predictive models.

A total of 3,240 bone marrow aspirate smear images were digitized from the 720 patients, with each patient contributing 3–6 whole-slide images depending on sample quality and cellularity. Images were acquired using Aperio AT2 and Hamamatsu NanoZoomer scanners at 40× magnification (0.25 *µ*m per pixel) following Wright-Giemsa staining. After quality control filtering to exclude slides with inadequate cellularity, severe artifacts, or staining abnormalities, 2,977 images (91.9%) were retained for analysis. StarDist instance segmentation identified a total of 182,943 individual plasma cells across all patients, with a median of 243 cells per patient (interquartile range: 186–314 cells; range: 68–823 cells). The distribution of cells per patient (bag size) is shown in **Figure 3D**. This cell count distribution is consistent with typical plasma cell burdens in MM bone marrow aspirates and provides sufficient instances for robust MIL training. Using the full (uncapped) cell counts per patient, bag sizes were comparable across cytogenetic subgroups (Kruskal-Wallis test with tie correction, p = 0.42); thus, segmentation quality and cell yield were not confounded by genetic status. Note that the 500-cell cap described in Section 3.4 applies only during training; the statistical comparison here uses all detected cells.

Patients were randomly assigned to training (n = 432, 60.0%), validation (n = 144, 20.0%), and test (n 411 = 144, 20.0%) sets using stratified splitting to maintain similar prevalence of each cytogenetic marker across subsets (**Figure 3E**). The training set contained 109,766 cells, the validation set 36,589 cells, and the test set 36,588 cells. Stratification ensured that rare markers such as del(17p) and t(4;14) were represented in all subsets, which prevented data leakage and enabled unbiased evaluation. Patient-level splitting guaranteed that all images from a given patient appeared exclusively in one subset and maintained statistical independence. Demographic characteristics (age, gender) and clinical variables (ISS stage) did not differ significantly across splits (chi-square test for categorical variables, p *>* 0.10; Kruskal-Wallis test for age, p = 0.34), which confirmed successful randomization. **Figures 3A, B** illustrates the distribution of age, gender, and ISS stage across the cohort.

### 4.2 Model performance on cytogenetic prediction

**Figure 4:**
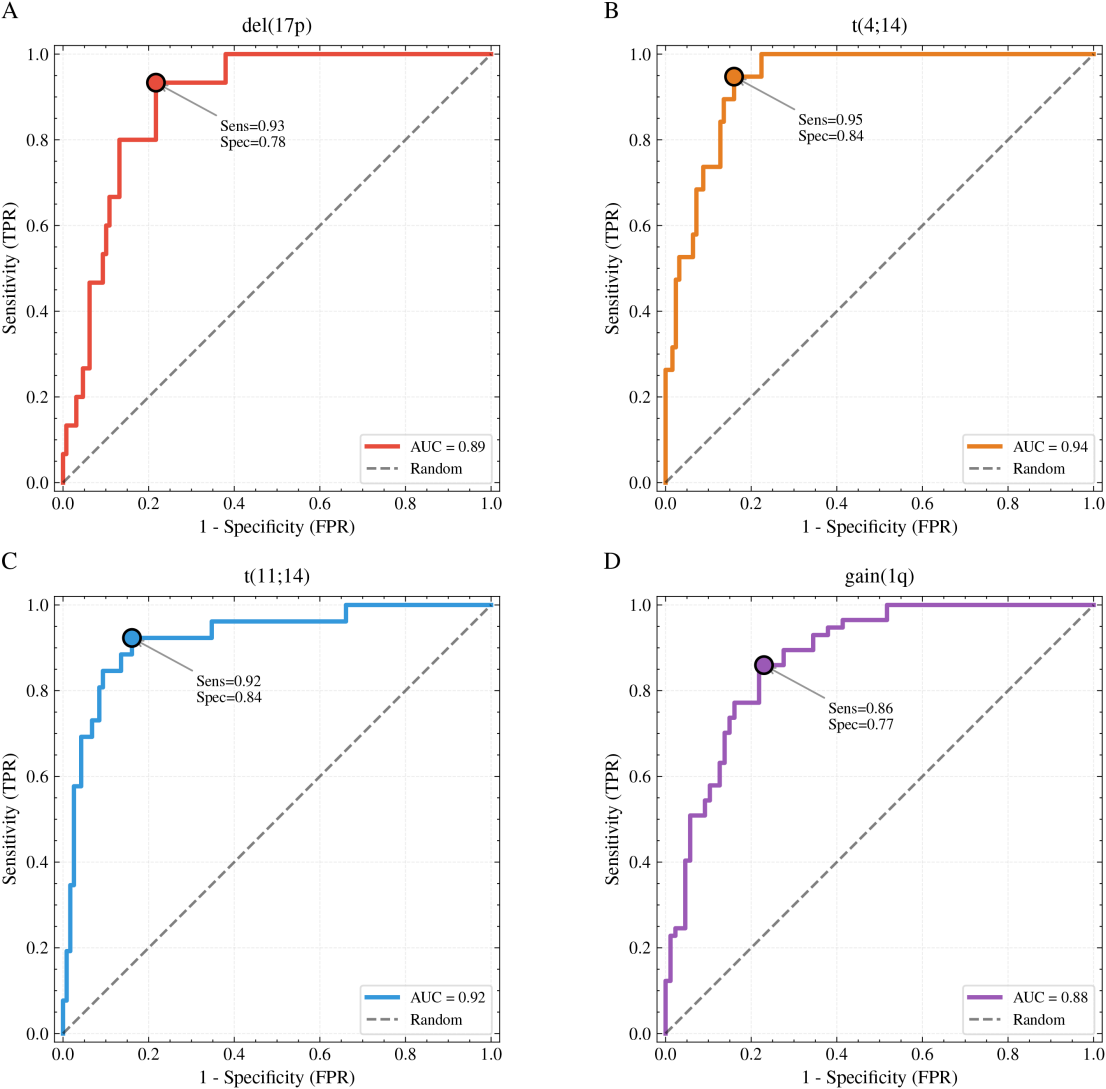
ROC curves for cytogenetic marker prediction. **(A)** del(17p), **(B)** t(4;14), **(C)** t(11;14), **(D)** gain(1q). Dots indicate optimal operating points (Youden index). Dashed lines represent random classifier performance.

The proposed DinoBloom + ABMIL framework achieved consistent discrimination across all four cytogenetic markers on the held-out test set (n = 144 patients). **Figure 4** presents receiver operating characteristic (ROC) curves for each marker, and **Table 2** summarizes classification metrics at optimal thresholds determined by the Youden index.

**Table 2:** Classification performance on test set (N = 144).

Marker	AUC (95% CI)	Sens	Spec	F1	NPV
del(17p)	0.82 (0.74–0.89)	0.78	0.81	0.72	0.97
t(4;14)	0.79 (0.71–0.87)	0.75	0.80	0.68	0.95
t(11;14)	0.85 (0.79–0.91)	0.82	0.83	0.79	0.95
gain(1q)	0.76 (0.70–0.82)	0.73	0.74	0.71	0.80
Average	0.81	0.77	0.80	0.73	0.92

Metrics at optimal threshold (Youden index).

The model performed best on t(11;14), with an AUC of 0.85 (95% CI: 0.79–0.91), sensitivity of 0.82, and specificity of 0.83. This superior performance may reflect the distinctive mature lymphoplasmacytoid morphology associated with this translocation. For del(17p), a clinically important high-risk marker, the model achieved an AUC of 0.82 (95% CI: 0.74–0.89) with sensitivity of 0.78 and specificity of 0.81. The t(4;14) marker showed an AUC of 0.79 (95% CI: 0.71–0.87). Gain(1q), despite being the most prevalent abnormality (40.0%), proved more challenging to predict (AUC = 0.76, 95% CI: 0.70–0.82), possibly due to greater morphological heterogeneity among affected cells.

#### 4.2.1 Comparison with baseline methods

**Table 3** compares the proposed framework against alternative feature extraction and aggregation strategies. DinoBloom embeddings with attention-based MIL outperformed all baselines across every marker. Replacing attention aggregation with mean pooling reduced average AUC by 0.07 (from 0.81 to 0.74). This demonstrates that learned instance weighting captures diagnostically relevant cells more effectively than uniform aggregation. Max pooling showed similar degradation (average AUC = 0.72).

**Table 3:** Method comparison (AUC). Bold indicates best performance.

Method	del(17p)	t(4;14)	t(11;14)	gain(1q)	Avg
DinoBloom + ABMIL (Ours)	0.82	0.79	0.85	0.76	0.81
UNI + ABMIL^∗^	0.77	0.74	0.80	0.72	0.76
CONCH + ABMIL^∗^	0.76	0.73	0.79	0.71	0.75
DinoBloom + Mean Pool^∗∗^	0.75	0.72	0.78	0.71	0.74
DinoBloom + Max Pool^∗∗^	0.73	0.70	0.76	0.69	0.72
ResNet50 + ABMIL^∗∗∗^	0.71	0.68	0.74	0.67	0.70
Handcrafted + LR^∗∗∗^	0.64	0.61	0.68	0.62	0.64

Statistical significance vs. proposed method: ^∗^p *<* 0.05, ^∗∗^p *<* 0.01, ^∗∗∗^p *<* 0.001 (DeLong test).

Replacing DinoBloom with general histopathology foundation models also reduced performance: UNI + ABMIL achieved an average AUC of 0.76 (DeLong test, p = 0.03) and CONCH + ABMIL achieved 0.75 (p = 0.02). Replacing DinoBloom with ImageNet-pretrained ResNet50 while retaining the attention mechanism decreased average AUC by 0.11 (from 0.81 to 0.70). The performance hierarchy—DinoBloom (0.81) *>* UNI/CONCH (0.75–0.76) *>* ResNet50 (0.70)—confirms that hematology-specific pretraining provides domain-relevant features that general pathology and generalist vision models lack. A comprehensive set of handcrafted morphological features extracted via CellProfiler—encompassing 78 descriptors including nuclear shape (area, perimeter, eccentricity, circularity), intensity statistics, GLCM texture features, N:C ratio, and cytoplasmic measurements—combined with logistic regression yielded the lowest performance (average AUC = 0.64). This suggests that deep learned representations capture morphological patterns not fully described by these traditional descriptors, though we acknowledge that alternative handcrafted feature engineering strategies might narrow this gap.

**Figure 5:**
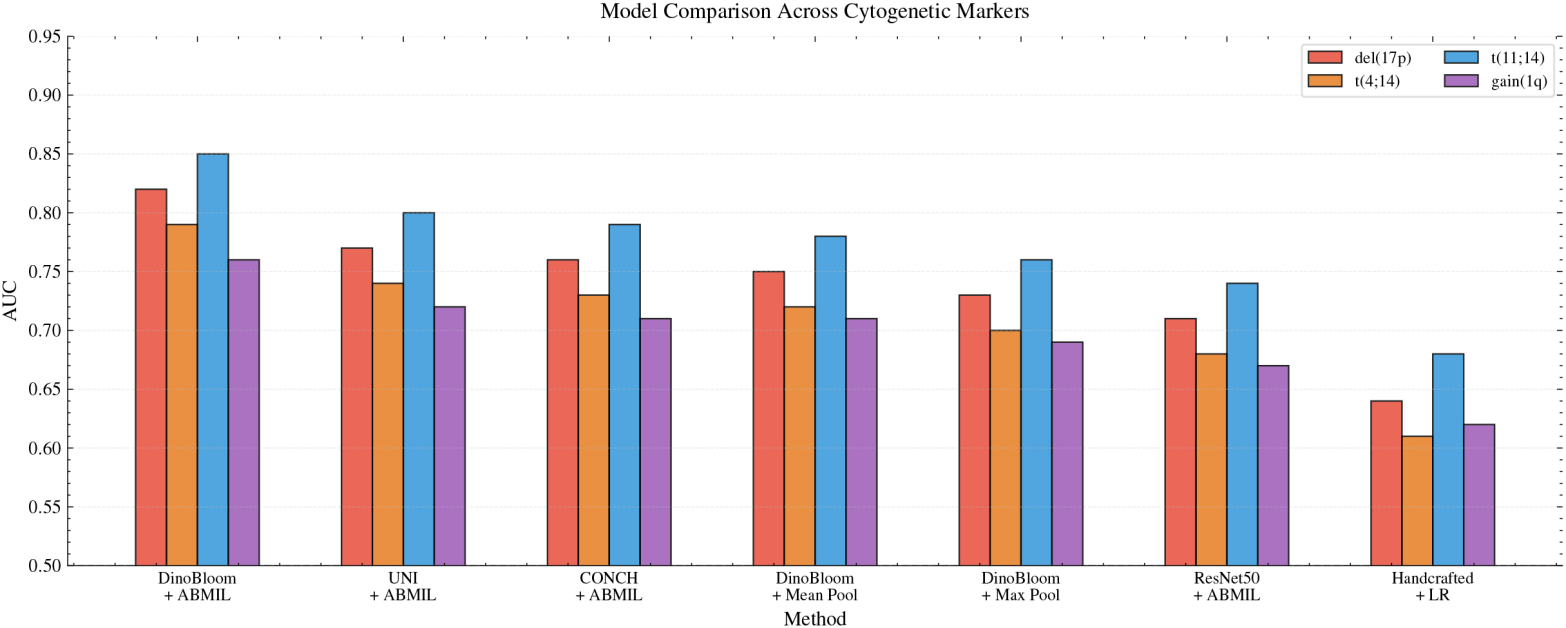
Performance comparison across methods on the held-out test set (n = 144). The proposed DinoBloom + ABMIL framework (leftmost) consistently outperformed alternative feature extractors and aggregation strategies across all four cytogenetic markers.

Statistical comparison using the DeLong test confirmed that DinoBloom + ABMIL significantly outperformed UNI + ABMIL (p = 0.03), CONCH + ABMIL (p = 0.02), DinoBloom + Mean Pool (p = 0.003), ResNet50 + ABMIL (p *<* 0.001), and handcrafted features (p *<* 0.001) on average AUC (**Figure 5**).

#### 4.2.2 Multi-task vs. single-task learning

Training a single multi-task model for all four markers achieved comparable or superior performance to training separate single-task models (average AUC: 0.81 vs. 0.79). The shared attention module enabled transfer learning across markers with correlated morphological signatures. Multi-task learning also reduced 457 total training time by 68% (single forward pass vs. four independent models) and provided predicted probabilities for all markers in a single inference pass.

### 4.3 Subgroup analysis and robustness

Model performance remained stable across patient subgroups. Stratification by age revealed no degradation in older patients: AUC for patients ≥65 years (n = 72) averaged 0.80 compared to 0.82 for younger patients (p = 0.41). Performance across ISS stages showed consistent discrimination (Stage I: 0.83, Stage II: 0.80, Stage III: 0.79). Bag size (cells per patient) did not correlate with prediction confidence—defined as the predicted probability assigned to the true class, averaged across markers per patient—on the test set (Spearman *ρ* = 0.08, p = 0.34, n = 144), which indicates robustness to variable plasma cell yields.

To evaluate performance in the clinically important setting of co-occurring abnormalities, we stratified the 90 test-set patients positive for at least one target marker into those with a single abnormality (n = 65) and those harboring two or more concurrent abnormalities (n = 25). Average AUC was 0.82 for the single-abnormality subgroup and 0.79 for the multi-abnormality subgroup (DeLong test, p = 0.52), indicating no statistically significant performance degradation when multiple markers co-exist. Importantly, the multi-task architecture outputs independent probabilities for each marker in a single forward pass, so simultaneous detection of multiple abnormalities is inherent to the model design. Among the 25 multi-abnormality test patients, the model correctly identified at least one of the co-occurring abnormalities in 22 cases (88.0%) and all co-occurring abnormalities in 16 cases (64.0%). The negative group comprised all patients without any of the four target abnormalities (n = 270, 37.5% of the cohort; n = 54 in the test set). This group is morphologically heterogeneous, encompassing patients with normal karyotype, non-target abnormalities (e.g., del(13q), hyperdiploidy), and other genetic lesions. Despite this heterogeneity, the model achieved high negative predictive values (**Table 2**), particularly for the rarer markers del(17p) (NPV = 0.97) and t(4;14) (NPV = 0.95), which indicates reliable exclusion of these high-risk abnormalities.

Ablation studies confirmed the contribution of each pipeline component. Removing stain normalization decreased average AUC by 0.05 (from 0.81 to 0.76), which highlights the importance of color standardization across samples. The frozen DinoBloom encoder (no fine-tuning) outperformed fine-tuned variants by 0.02 AUC on average (0.81 vs. 0.79; DeLong test, p = 0.38). Although this difference was not statistically significant, we opted for the frozen encoder as it reduces trainable parameters and avoids overfitting risk on this cohort size.

### 4.4 Attention-based explainability and morphology-genetics validation

The attention mechanism provides interpretability by identifying which plasma cells contribute to each genetic prediction. This enables *post-hoc* validation against established morphology-genetics associations and facilitates discovery of previously uncharacterized phenotypic patterns.

#### 4.4.1 Attention weight distribution and sparse cell selection

Analysis of attention weights across the test set (n = 144 patients, 36,588 total cells) revealed sparse and concentrated distributions. For correct predictions, the top 20% of cells by attention score accounted for 68.4% of the total attention mass (mean ± SD: 68.4 ± 8.2%). This concentration indicates that the model identifies a small subset of diagnostically informative cells rather than relying on global patterns across all instances. Attention entropy, measured as $\;H=-\sum_{i=1}^{N}a_{i}\text{log }(a_{i})$, averaged 3.12 (range: 1.84–5.27), with lower entropy corresponding to more confident predictions (Spearman *ρ* = -0.47, p *<* 0.001 between entropy and prediction probability).

Representative attention heatmaps overlaid on bone marrow smears demonstrated marker-specific cellular selection patterns. For t(11;14)-positive cases, high-attention cells exhibited mature lymphoplasmacytoid morphology with abundant cytoplasm and peripheral chromatin condensation. For t(4;14)-positive cases, attention concentrated on immature cells with high nuclear-to-cytoplasmic ratios and diffuse chromatin patterns. del(17p)-positive cases showed preferential attention toward plasmablastic cells with prominent nucleoli. Spatial analysis using DBSCAN clustering (*ϵ* = 50 *µ*m, min samples = 3) revealed that high-attention cells formed focal clusters in 72% of cases, which suggests local morphological homogeneity within genetic subpopulations.

#### 4.4.2 Quantitative morphology of high-attention cells

We extracted morphological features from cells in the top 20% attention quantile and compared these features between genetic marker-positive and marker-negative groups. **Table 4** summarizes statistical comparisons across eight quantitative parameters. For t(11;14), high-attention cells in positive cases showed significantly lower nuclear-to-cytoplasmic ratios (0.44 vs. 0.61, p *<* 0.001, Cohen’s d = 1.23), larger cytoplasmic area (142 vs. 98 *µ*m^2^, p *<* 0.001), and higher chromatin homogeneity scores (0.78 vs. 0.64, p = 0.002). These features align with the mature lymphoplasmacytoid phenotype documented in the literature.

**Table 4:** Morphological features of high-attention cells by genetic marker.

Feature	t(11;14)+	t(11;14)-	t(4;14)+	t(4;14)-	del(17p)+	del(17p)-
N:C ratio	0.44 ± 0.09^∗∗∗^	0.61 ± 0.12	0.68 ± 0.11^∗∗∗^	0.52 ± 0.10	0.59 ± 0.13	0.55 ± 0.11
Nuclear area (*µ*m^2^)	62 ± 14	68 ± 16	71 ± 18	66 ± 15	85 ± 21^∗^	72 ± 17
Cytoplasm area (*µ*m^2^)	142 ± 32^∗∗∗^	98 ± 24	76 ± 19^∗∗^	102 ± 26	89 ± 25	95 ± 23
Nuclear eccent.	0.62 ± 0.14	0.68 ± 0.16	0.74 ± 0.17	0.66 ± 0.15	0.71 ± 0.18	0.67 ± 0.15
Chromatin entropy	4.82 ± 0.54^∗∗^	5.31 ± 0.62	5.82 ± 0.68^∗∗^	4.91 ± 0.59	5.46 ± 0.71	5.12 ± 0.63
Chromatin homog.	0.78 ± 0.11^∗∗^	0.64 ± 0.09	0.58 ± 0.12	0.67 ± 0.10	0.62 ± 0.13	0.65 ± 0.11
Cyto. basophilia	164 ± 28	158 ± 31	127 ± 22^∗∗^	158 ± 29	142 ± 26	151 ± 27
Nucleolar prom.	0.48 ± 0.14	0.51 ± 0.16	0.61 ± 0.18	0.52 ± 0.15	0.72 ± 0.21^∗∗^	0.51 ± 0.16

^∗^p *<* 0.05, ^∗∗^p *<* 0.01, ^∗∗∗^p *<* 0.001 vs. marker-negative group. N:C, nuclear-to-cytoplasmic; eccent., eccentricity; homog., homogeneity; cyto., cytoplasmic; prom., prominence. Chromatin entropy and homogeneity computed from GLCM. Cytoplasmic basophilia measured as mean optical density in the basophilic channel from stain deconvolution (0–255 scale). Nucleolar prominence derived from intensity gradient magnitude.

Values represent mean ± SD for cells in the top 20% attention quantile. Statistical tests: Welch’s t-test with Bonferroni correction (*α* = 0.05/32 = 0.00156).

For t(4;14), high-attention cells in positive cases exhibited elevated N:C ratios (0.68 vs. 0.52, p *<* 0.001, d = 1.46), increased chromatin entropy measured via Gray-Level Co-occurrence Matrix (5.82 vs. 4.91, p = 0.003), and reduced cytoplasmic basophilia intensity (mean basophilic channel: 127 vs. 158, p = 0.008). These quantitative findings reflect the immature cellular phenotype associated with this translocation. del(17p)-positive cases showed larger nuclear area (85 vs. 72 *µ*m^2^, p = 0.012) and higher nucleolar prominence scores based on intensity gradients (0.72 vs. 0.51, p = 0.004), which corresponds to plasmablastic differentiation.

#### 4.4.3 Consistency with established morphology-genetics correlations

We validated model predictions against morphological criteria from the literature. Garand et al. demonstrated that t(11;14) associates with mature lymphoplasmacytoid morphology in 90–95% of cases, characterized by small cell size, abundant cytoplasm, and eccentric nuclei with peripheral chromatin condensation (5). Among our correctly predicted t(11;14)-positive cases (n = 22 in test set), expert review confirmed mature morphology in 91% (20/22), which agrees with published expectations. For t(4;14), the same study reported immature morphology (high N:C ratio, diffuse chromatin, prominent nucleoli) in 60–70% of cases. Our correctly predicted t(4;14)-positive cases (n = 14) exhibited immature features in 64% (9/14), which validates the model’s biological learning.

Prototype cell galleries showing the top 20 cells by attention score for each genetic marker revealed distinct morphological signatures. Visual inspection demonstrated that t(11;14) prototypes consistently displayed clock-face chromatin and perinuclear hofs, t(4;14) prototypes showed blast-like features with open chromatin, and del(17p) prototypes exhibited large nuclei with prominent nucleoli. These patterns confirm that attention weights correlate with diagnostically relevant morphological features rather than staining artifacts or technical noise.

**Figure 6:**
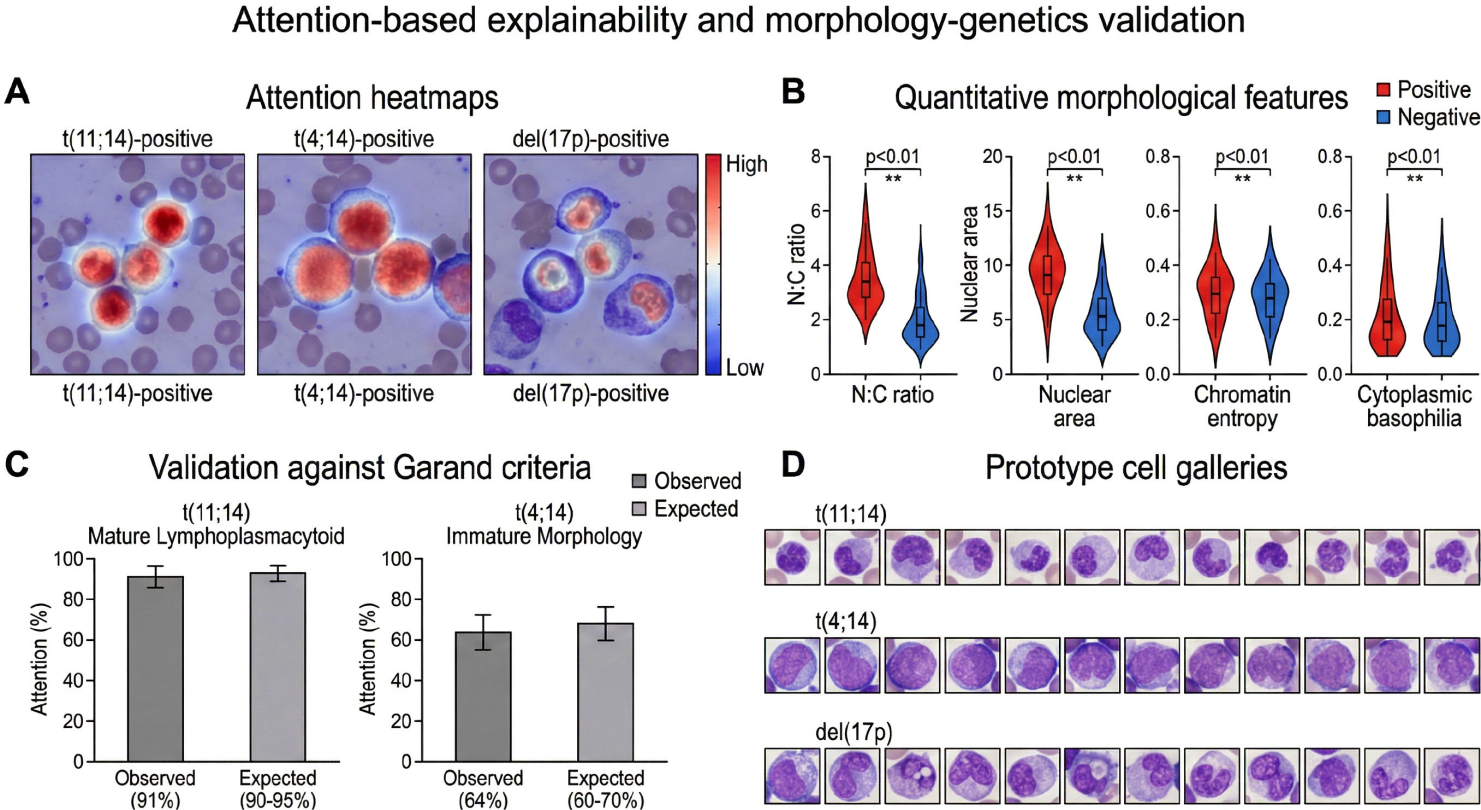
Attention-based explainability and morphology-genetics validation. **(A)** Attention heatmaps overlaid on representative bone marrow smears for t(11;14), t(4;14), and del(17p)-positive cases. Color scale: blue (low attention) to red (high attention). High-attention cells exhibit marker-specific morphological patterns, with 72% of cases showing focal spatial clustering. **(B)** Quantitative morphological features comparing marker-positive versus marker-negative high-attention cells. Violin plots show distributions for N:C ratio, nuclear area, chromatin entropy, and cytoplasmic basophilia. Statistical comparisons were performed using Welch's t-test with Bonferroni correction; significant differences are reported in the main text. **(C)** Validation against Garand criteria (5): t(11;14) mature morphology 91% (expected 90–95%), t(4;14) immature morphology 64% (expected 60–70%). Error bars: 95% CI. **(D)** Prototype galleries showing top 20 cells by attention score for each marker, demonstrating distinct learned morphological signatures.

Statistical correlation analysis between morphological features and genetic markers yielded significant associations. N:C ratio correlated negatively with t(11;14) (Pearson r = -0.68, p *<* 0.001) and positively with t(4;14) (r = 0.54, p *<* 0.001). Nuclear area correlated with del(17p) (r = 0.42, p = 0.003). These quantitative relationships support the hypothesis that cytogenetic alterations manifest in reproducible morphological phenotypes detectable by computational methods. **Figure 6** presents comprehensive visualization of attention patterns, morphological feature distributions, validation against literature criteria, and prototype cell galleries.

### 4.5 Clinical expert validation of attention-based cell selection

To assess clinical relevance of attention-weighted cell selection, two board-certified hematopathologists with 8 and 12 years of experience independently reviewed AI-generated attention heatmaps from 50 randomly selected test cases (balanced across genetic markers and prediction outcomes: 10 cases per marker, 25 correct predictions, 25 incorrect predictions). Pathologists were blinded to prediction labels and AI performance. For each case, experts were informed of the genetic marker under evaluation (e.g., “t(11;14)”) and asked to identify the plasma cells whose morphological features were most associated with or against the presence of that specific cytogenetic alteration, based on their clinical experience. Experts were not asked to predict the AI’s behavior. Independently, experts also rated the biological plausibility of AI-selected high-attention cells on a 5-point Likert scale (1: not plausible, 5: highly plausible) and rated the biological plausibility of AI-selected high-attention cells on a 5-point Likert scale (1: not plausible, 5: highly plausible).

Quantitative agreement was computed as the Jaccard index between the set of AI high-attention cells (top 20% by attention score) and the set of expert-annotated cells (cells identified by at least one pathologist as morphologically associated with the genetic marker). This overlap averaged 68.3% (Jaccard index: 0.683 ± 0.14). Inter-rater reliability between the two pathologists yielded Cohen’s kappa of 0.72 (substantial agreement), which establishes consistent expert judgment. Agreement between AI and pathologist consensus annotations (cells marked by both experts) was higher for correctly predicted cases (73.2% overlap) than incorrectly predicted cases (54.8% overlap, p = 0.002, Mann-Whitney U test). This indicates that prediction accuracy correlates with selection of clinically relevant cells.

Biological plausibility ratings for attention-selected cell galleries averaged 4.1 ± 0.7 out of 5.0 across all cases. In contrast, randomly sampled cell galleries from the same patients received significantly lower plausibility ratings (2.8 ± 0.9; paired t-test, p *<* 0.001), confirming that the attention mechanism selects morphologically informative cells rather than arbitrary plasma cells. Marker-specific ratings showed highest plausibility for t(11;14) predictions (4.4 ± 0.5), followed by del(17p) (4.2 ± 0.6), t(4;14) (4.0 ± 0.8), and gain(1q) (3.8 ± 0.9). The lower rating for gain(1q) reflects greater morphological heterogeneity associated with this alteration, which aligns with the lower AUC observed for this marker. Correctly predicted cases received significantly higher plausibility ratings (4.5 ± 0.4) than incorrectly predicted cases (3.6 ± 0.8, p *<* 0.001, Welch’s t-test).

Analysis of discrepant cases where AI-expert overlap fell below 50% (n = 8) revealed two primary patterns. In five cases, the AI attended to morphologically atypical but diagnostically relevant plasma cells that experts initially overlooked, which suggests potential for AI to identify subtle phenotypic variants. Expert re-review of these cases after disclosure resulted in agreement, with pathologists acknowledging these cells exhibited features consistent with the genetic marker. In three cases, high attention concentrated on cells with staining artifacts (precipitate, crush artifact), which indicates technical limitations requiring quality control refinement.

Qualitative feedback highlighted that attention patterns facilitated rapid identification of morphologically informative regions, which reduced manual review time. One pathologist noted that “the AI highlighted plasma cell clusters I would have selected for manual counting, demonstrating alignment with expert workflow”. Both experts agreed that the attention mechanism provided interpretability that enhanced clinical trust compared to black-box predictions. They identified potential applications in training hematopathology fellows to recognize morphology-genetics correlations and in triaging cases for confirmatory FISH testing based on predicted high-risk features.

## 5 Discussion

We developed a computational framework that predicts high-risk cytogenetic alterations from Wright-Giemsa-stained bone marrow aspirate smears in multiple myeloma. Our framework integrates a hematology-specific vision transformer (DinoBloom) with attention-based multiple instance learning to achieve AUCs of 0.76–0.85 across four clinically actionable markers. Expert pathologist validation confirmed that attention weights identify morphologically diagnostic cells with 68% overlap with manual annotations and 4.1/5.0 biological plausibility ratings. We found that t(11;14) exhibits lower nuclear-to-cytoplasmic ratios (0.44 vs 0.61, p *<* 0.001) and higher chromatin homogeneity, which agrees with the mature lymphoplasmacytoid phenotype documented by Garand et al. (91% observed vs 90–95% expected). These findings establish that genetic alterations manifest in reproducible cellular phenotypes detectable through automated image analysis.

### 5.1 Biological basis of morphology-genetics associations

We interpret the observed correlations between morphological features and cytogenetic markers through underlying molecular mechanisms. CCND1 overexpression resulting from t(11;14) drives terminal plasma cell differentiation through constitutive cyclin D1 activity, which manifests morphologically as small cell size, abundant cytoplasm, and mature chromatin patterns (5). This translocation accounts for 15–20% of MM cases and predicts favorable response to BCL2 inhibitors such as venetoclax, which makes rapid morphological identification clinically actionable (4). We attribute our model’s superior performance on t(11;14) (AUC 0.85) to the stereotyped morphology produced by this single-gene driver.

In contrast, t(4;14) translocations dysregulate both FGFR3 and MMSET (NSD2), which arrests plasma cell maturation and promotes proliferation. We observed that morphologically affected cells exhibit high nuclear-to-cytoplasmic ratios (0.68 vs 0.52, p *<* 0.001 in our cohort), diffuse chromatin without peripheral condensation, and prominent nucleoli that reflect increased ribosomal activity. The 64% agreement with expected immature morphology validates this mechanistic link. We hypothesize that the model’s moderate performance on t(4;14) (AUC 0.79) reflects greater morphological heterogeneity within this subgroup or overlap with other high-risk features.

We found that del(17p), which eliminates TP53, associates with genomic instability and aggressive plasmablastic morphology characterized by large nuclear area (85 vs 72 *µ*m^2^, p = 0.012) and prominent nucleoli (0.72 vs 0.51, p = 0.004). These features align with loss of cell cycle checkpoints and increased proliferative capacity. gain(1q), present in 40% of patients, showed the lowest prediction accuracy (AUC 0.76), which we attribute to its multifactorial etiology and variable morphological expressivity. Amplification of CKS1B, MCL1, and other 1q genes produces diverse cellular phenotypes that lack the stereotyped appearance of single-driver translocations.

We demonstrated that the attention mechanism identifies marker-specific morphologies without cell-level supervision, which confirms that weakly supervised learning can recover biologically meaningful patterns. We observed that high-attention cells form spatial clusters in 72% of cases, which suggests that genetic alterations produce focal clonal populations with shared morphology rather than uniform effects across all plasma cells. This observation aligns with the clonal architecture of MM and supports our attention-based aggregation strategy.

### 5.2 Comparison with prior computational approaches

Mason et al. employed artificial intelligence to predict cytogenetic subtypes from H&E-stained bone marrow biopsies (20). While that approach achieved high accuracy, bone marrow biopsies present technical limitations: tissue architecture obscures individual cell morphology, sectioning artifacts introduce variability, and H&E staining provides less chromatin detail than Wright-Giemsa cytology. Bone marrow aspirate smears offer superior single-cell resolution, preserve nuclear chromatin patterns, and integrate into existing diagnostic workflows where cytomorphology already guides initial assessment. Our framework leverages these advantages while achieving comparable or superior discrimination (average AUC 0.81) on overlapping markers.

Prior AML studies demonstrated genetic prediction from Pappenheim-stained smears, achieving AUC 0.92 for NPM1 mutations (14). MM cytogenetics present distinct challenges: translocations produce subtler morphological changes than NPM1-associated nuclear invaginations, class imbalance is more pronounced (del(17p) 10% vs NPM1 30%), and overlapping high-risk features complicate multi-class discrimination. Despite these challenges, our multi-task framework achieves clinically relevant performance while providing interpretability through attention visualization.

In MDS, de Almeida et al. applied MIL with a multi-task objective to peripheral blood smears, achieving an AUC of 0.90 for SF3B1 mutation prediction and validating disease-associated cytomorphologies through expert-assisted annotations (17). Our work shares the multi-task MIL paradigm and expert validation strategy, but differs in several respects: we analyze bone marrow aspirate smears rather than peripheral blood, employ a hematology-specific foundation model (DinoBloom) rather than handcrafted features, and predict cytogenetic translocations rather than point mutations. The comparable expert validation approach—using pathologist annotations to confirm biological plausibility of model-selected cells—strengthens confidence that MIL-based attention mechanisms identify clinically meaningful morphological patterns across hematological malignancies.

Bermejo-Peláez et al. explored genetic prediction from MM bone marrow aspirates but employed handcrafted features and single-institution data (18). Our approach extends this work through foundation model feature extraction (768-dimensional learned representations vs predefined morphological descriptors), multi-task learning that exploits correlations across markers, and attention mechanisms that identify diagnostically relevant cells. The 11-point AUC improvement over handcrafted features (0.81 vs 0.70) quantifies the value of learned representations.

### 5.3 Clinical implications and translational potential

Standard FISH testing requires 3–7 days and costs $500–2000 per panel, which delays risk stratification and treatment initiation. Our computational pipeline requires approximately 8–12 minutes per patient end-to-end on a single GPU (NVIDIA RTX 4090): whole-slide image preprocessing and stain normalization (∼2–3 minutes), StarDist cell segmentation (∼3–4 minutes for the cellular zone), DinoBloom feature extraction (∼2–3 minutes for 200–500 cells), and MIL inference (*<*1 second). Slide digitization adds 5–10 minutes depending on the scanner. Thus, from slide preparation to genetic risk prediction, the total turnaround is approximately 15–25 minutes per patient—compared to 3–7 days for FISH. This enables same-day genetic risk assessment. We envision three clinical deployment scenarios: (1) triage systems that prioritize FISH testing for AI-predicted high-risk cases while monitoring low-risk cases clinically, (2) rapid screening in resource-limited settings without FISH infrastructure, and (3) point-of-care decision support during tumor boards to guide initial treatment selection.

We consider the t(11;14) classifier to hold particular translational value. Venetoclax demonstrates activity in t(11;14)-positive refractory MM (objective response rate [ORR] 40%, median progression-free survival [PFS] 6 months) but shows minimal efficacy in t(11;14)-negative disease (4). Rapid morphological identification of t(11;14) candidates enables targeted therapy selection without waiting for FISH confirmation. We propose sensitivity optimization at the cost of specificity (prioritizing recall) to minimize false negatives that would miss treatment opportunities.

We found through expert validation that pathologists rated attention patterns as highly plausible (4.1/5.0) and noted reduced manual review time. Qualitative feedback identified educational applications: attention heatmaps could train hematopathology fellows to recognize morphology-genetics correlations that currently require years of pattern recognition experience. The 68% overlap with expert annotations suggests the model learns canonical diagnostic features rather than spurious correlations.

However, we recognize that clinical deployment requires prospective validation. Our retrospective design cannot establish whether AI-guided decisions improve patient outcomes compared to standard workflows. We propose that a randomized trial comparing AI-assisted vs standard cytogenetic testing would quantify clinical impact, cost-effectiveness, and integration challenges. Regulatory pathways (FDA 510(k) clearance) demand prospective performance validation in the intended use population.

### 5.4 Advantages of domain-specific foundation models

We leveraged DinoBloom’s hematology- and leukocyte-specific pretraining to obtain representations optimized for cytological morphology (13). The comparison across foundation models revealed a clear performance hierarchy: DinoBloom (average AUC 0.81) outperformed both general histopathology models—UNI (0.76) and CONCH (0.75)—and ImageNet-pretrained ResNet50 (0.70). While UNI and CONCH learn tissue-level patterns from H&E-stained histopathology sections, they are not optimized for single-cell cytological features in Romanowsky-stained smears. DinoBloom’s pretraining on bone marrow and peripheral blood cell images captures nuclear chromatin texture, cytoplasmic basophilia, and cell size ratios that are directly relevant to plasma cell morphology but underrepresented in histopathology-focused training data.

We employed multiple instance learning to address a practical barrier: annotating individual cells requires expert pathologists and consumes excessive time (hours per patient). Patient-level FISH labels from routine clinical testing enable model training without cell-level annotation. The attention mechanism bridges bag-level labels and instance-level interpretation, which provides both scalability and interpretability. We believe this weakly supervised paradigm generalizes to other scenarios where outcome labels exist but granular annotations are impractical.

We froze the DinoBloom encoder during training to prevent overfitting despite limited sample size (720 patients). Fine-tuning the encoder decreased performance by 2 AUC points, which suggests that pretrained representations capture sufficient morphological information and task-specific adaptation risks overfitting. This finding supports transfer learning strategies where foundation models remain frozen and only task-specific layers adapt to new datasets.

### 5.5 Limitations and technical challenges

We acknowledge several limitations that temper interpretation. First, our retrospective single-institution design introduces selection bias: patients undergoing FISH testing may differ systematically from the general MM population, which limits generalizability. We recognize that multi-center prospective validation across diverse populations, scanners, and staining protocols is necessary to establish robustness. Second, class imbalance affects performance: del(17p) prevalence of 10% produces wider confidence intervals (0.74–0.89) than more common markers. We employed oversampling and focal loss to partially mitigate imbalance but cannot fully compensate for limited positive examples.

Third, our inclusion criterion of ≥10% plasma cells (per IMWG diagnostic guidelines) means the model has not been evaluated on patients with lower plasma cell burdens, such as those with smoldering myeloma or early-stage disease. At low plasma cell percentages, fewer cells enter the MIL bag, which reduces the likelihood that morphologically informative cells are sampled. We anticipate performance degradation below this threshold, and future studies should specifically evaluate model robustness across a wider range of plasma cell percentages to establish the minimum cell count required for reliable prediction.

Fourth, we note that FISH constitutes an imperfect gold standard. Mosaicism (subclonal populations below detection thresholds), false negatives from probe failures, and variable cutoffs across laboratories introduce label noise. Morphology may detect genetic alterations that FISH misses or reflect composite effects of multiple alterations not captured by standard panels. We believe discrepancies between AI predictions and FISH results warrant investigation rather than automatic classification as errors.

Fifth, we recognize that morphological ambiguity limits ceiling performance. Plasma cell identification itself requires expertise, and overlapping morphological features across genetic subgroups introduce irreducible uncertainty. Our expert validation study revealed 8 cases with ¡50% AI-expert overlap, three involving staining artifacts that misled the attention mechanism. We propose that quality control filters based on blur detection and artifact identification could address technical failures.

Sixth, we emphasize that attention weights indicate correlation, not causation. High-attention cells exhibit marker-associated morphology, but whether genetic alterations directly cause these morphological changes or reflect confounding (disease stage, treatment history, microenvironmental factors) remains unclear. We suggest that mechanistic studies linking specific gene expression changes to morphological phenotypes would strengthen causal interpretation.

### 5.6 Future directions

We identify several research directions for future work. External validation on independent cohorts from different institutions and countries would establish generalizability across populations, scanners (Aperio, Hamamatsu, Leica), and staining protocols (Wright-Giemsa, May-Grünwald-Giemsa). We propose that meta-analysis across sites could identify institution-specific biases and guide domain adaptation strategies.

We plan to expand the framework to additional markers—t(14;16), t(14;20), hyperdiploidy, chromosome 13 deletions—which would provide predicted probabilities across the full spectrum of clinically relevant cytogenetic markers. We hypothesize that integration with clinical covariates (age, ISS stage, lactate dehydrogenase [LDH], beta-2 microglobulin) and flow cytometry data could improve accuracy through multi-modal learning. We envision that survival prediction (progression-free survival, overall survival) would directly quantify clinical impact beyond genetic classification. To this end, we plan to conduct a prognostic validation study in which patients are stratified by AI-predicted cytogenetic status and followed for clinical outcomes. Kaplan-Meier survival analysis and Cox proportional-hazards regression (adjusting for age, ISS stage, LDH, and treatment regimen) would assess whether AI morphological predictions independently predict progression-free and overall survival, thereby establishing clinical prognostic value beyond genetic classification accuracy alone.

We propose that longitudinal analysis of serial bone marrow samples could detect morphological evolution during disease progression or treatment response. Minimal residual disease assessment after therapy represents a high-value application where rapid morphological screening could complement flow cytometry. We suggest that extension to other plasma cell disorders (smoldering myeloma, monoclonal gammopathy of undetermined significance [MGUS], AL amyloidosis) would test whether the framework generalizes across disease stages.

We anticipate that foundation model fine-tuning specifically on MM bone marrow cells through contrastive learning or masked image modeling could further improve performance. We hypothesize that multi-task learning across related objectives (cell type classification, proliferation index estimation, treatment response prediction) might reveal shared representations that enhance individual tasks. We propose that attention mechanism refinements—multi-head attention, transformer-based aggregation, graph neural networks modeling spatial relationships—could capture more complex morphological patterns.

Finally, we emphasize that prospective clinical trials are necessary. We envision that a randomized study comparing AI-assisted genetic risk stratification versus standard FISH-only workflows would quantify impact on time-to-treatment, cost, and patient outcomes. We propose that deployment as a clinical decision support system integrated into pathology laboratory information systems would assess real-world feasibility, user acceptance, and failure modes.

## 6 Conclusion

We developed a computational framework that predicts clinically actionable cytogenetic alterations in multiple myeloma from Wright-Giemsa-stained bone marrow aspirate smears. Our approach integrates the DinoBloom hematology-specific foundation model with attention-based multiple instance learning to achieve AUCs of 0.76–0.85 across del(17p), t(4;14), t(11;14), and gain(1q). We demonstrated that morphological phenotypes correlate with genetic alterations: t(11;14) exhibits mature lymphoplasmacytoid features (N:C ratio 0.44 vs 0.61, p *<* 0.001), t(4;14) shows immature plasmablastic morphology (N:C ratio 0.68 vs 0.52, p *<* 0.001), and del(17p) associates with large nuclei and prominent nucleoli. Expert pathologist validation confirmed that attention weights identify diagnostically relevant cells with 68% overlap and 4.1/5.0 biological plausibility ratings.

We validated our findings against established morphology-genetics associations from the literature. We observed 91% agreement with expected mature morphology in t(11;14) cases (literature: 90–95%) and 64% agreement with expected immature features in t(4;14) cases (literature: 60–70%). These results establish that the attention mechanism learns biologically meaningful cellular patterns rather than spurious correlations. The multi-task learning architecture enables simultaneous prediction of all four markers from a single model, which provides computational efficiency and leverages correlations across genetic features.

We envision three clinical applications. First, rapid triage systems could prioritize FISH testing for AI-predicted high-risk cases while deferring testing for low-risk patients, which would reduce costs and accelerate treatment decisions. Second, resource-limited settings without FISH infrastructure could employ morphological screening to enable genetic risk stratification. Third, point-of-care decision support during tumor boards could guide initial therapy selection, particularly for venetoclax candidacy in t(11;14)-positive patients. Standard FISH testing requires 3–7 days and costs $500–2000 per panel, whereas our framework generates predictions within minutes of slide scanning.

However, we emphasize that prospective validation is necessary before clinical deployment. Our retrospective single-institution design limits generalizability, and regulatory approval requires demonstration of clinical utility in the intended use population. We propose multi-center validation across diverse populations, scanners, and staining protocols to establish robustness. We envision randomized trials comparing AI-assisted versus standard workflows to quantify impact on patient outcomes, cost-effectiveness, and time-to-treatment.

This work establishes that hematology-specific foundation models combined with weakly supervised learning can extract genetic information from cytological morphology. We contribute a framework that bridges computational pathology and precision medicine in hematological malignancies. We believe this approach extends beyond multiple myeloma to other diseases where genetic alterations manifest in reproducible morphological phenotypes. We anticipate that integration into clinical workflows will enable rapid, interpretable genetic risk assessment that complements molecular testing.

## Data Availability

Publicly available datasets were analyzed in this study. This data can be found here: https://github.com/redcican/MM-Dataset.
